# GRB10 is a novel factor associated with gastric cancer proliferation and prognosis

**DOI:** 10.18632/aging.204603

**Published:** 2023-03-23

**Authors:** Li-Li Ren, Zhi-Wen Wang, Ren Sen, Zhou-Tong Dai, Xing-Hua Liao, Li-Juan Shen

**Affiliations:** 1 School of Food and Drug, Shenzhen Polytechnic, Guangdong 518055, China; 2 Institute of Biology and Medicine, College of Life and Health Sciences, Wuhan University of Science and Technology, Hubei 430081, China; 3 Clinical Academy, Changsha Health Vocational College, Hunan 410100, China; 4 Department of Obstetrics and Gynecology, Tongji Hospital, Tongji Medical College, Huazhong University of Science and Technology, Wuhan 430030, China; 5 Longgang District People's Hospital of Shenzhen, Guangdong 518172, China

**Keywords:** GRB10, gastric cancer, proliferation, prognosis

## Abstract

GRB10 and its family members GRB7 and GRB14 were important adaptor proteins. They regulated many cellular functions by interacting with various tyrosine kinase receptors and other phosphorus-containing amino acid proteins. More and more studies have shown that the abnormal expression of GRB10 is closely related to the occurrence and development of cancer. In our current research, expression data for 33 cancers from the TCGA database was downloaded for analysis. It was found that GRB10 was up-regulated in cholangiocarcinoma, colon adenocarcinoma, head and neck squamous carcinoma, renal chromophobe, clear renal carcinoma, hepatocellular carcinoma, lung adenocarcinoma, lung squamous carcinoma, gastric adenocarcinoma and thyroid carcinoma. Especially in gastric cancer, the high GRB10 expression was closely associated with poorer overall survival. Further research showed that the knockdown of GRB10 inhibited proliferation and migration ability in gastric cancer. Also, there was a potential binding site for miR-379-5p on the 3′UTR of GRB10. Overexpression of miR-379-5p in gastric cancer cells reduced GRB10-regulated gastric cancer proliferation and migration capacity. In addition, we found that tumor growth was slower in a mice xenograft model with knock down of GRB10 expression. These findings suggested that miR-379-5p suppresses gastric cancer development by downregulating GRB10 expression. Therefore, miR-379-5p and GRB10 were expected to be potential targets for the treatment of gastric cancer.

## INTRODUCTION

Gastric cancer is one of the common malignant tumors of the digestive system [1]. Studies have shown that the current 5-year survival rate after surgery for early gastric cancer is >90%. The 5-year overall survival rate after surgery for mid-stage gastric cancer is about 40% to 50%. The 5-year survival rate for patients with advanced gastric cancer after surgery is still lower than 30% [2]. However, early gastric cancer lacks specific clinical manifestations. 80% of patients were diagnosed in the middle and late stages [3]. The gold standard for diagnosing gastric cancer is still pathological biopsy under gastroscopy [4]. However, because the inspection method requires advanced equipment and specialized operators, the cost is also high, and it is not suitable for repeated inspections and large-scale population censuses. Although tumor markers and gastric function tests help identify patients with gastric cancer, their sensitivity and specificity vary significantly among individuals. They have not been used as early screening methods for gastric cancer [5]. Traditional screening of tumor markers has low efficiency due to the long experimental period. The advent of bioinformatics has dramatically increased the speed of screening for tumor markers. The studies have demonstrated that bioinformatics screening for tumor markers is a reliable method [6–8]. Therefore, bioinformatics is expected to screen new gastric cancer markers more quickly with excellent specificity and sensitivity.

Growth factor receptor-binding protein 10 (GRB10) is a member of the adaptor protein superfamily [9]. It is widely expressed in mammalian tissues, but its expression is not uniform [10]. In previous studies, GRB10 was mainly considered to be closely related to the insulin signaling pathway involved in the negative regulation of insulin/IGF signaling [11]. However, recent *in vitro* and *in vivo* studies have shown that GRB10 is also involved in regulating the occurrence and development of cancer, including regulating cell metabolism, cell growth and apoptosis [12, 13]. In acute myeloid leukemia, the expression of GRB10 is elevated, and overexpression of GRB10 in cells leads to abnormal cell proliferation [14]. Likewise, GRB10 is regarded as an oncogene in GBM and GSCs [15]. However, the expression of GRB10 in gastric cancer and its role is still unclear.

Non-coding RNAs are a class of RNAs without protein-coding functions, including long non-coding RNAs, microRNAs, and circular RNAs. Among them, miRNA is a non-coding RNA with a length of about 20–25 nt [16]. It binds to the 3′UTR region of target genes to silence gene expression. To achieve post-transcriptional regulation of target genes [17], more and more research results show that the abnormal expression of miRNA regulates the occurrence and development of gastric cancer [18]. However, whether miR-379-5p is involved in GRB10-mediated regulation of gastric cancer has not been elucidated.

In this study, we found that the expression of GRB10 was elevated in gastric cancer using bioinformatics combined experiments in the present study. In addition, the effect of GRB10 on the biological behavior of gastric cancer cell proliferation and migration ability was investigated. On this basis, an effective intervention strategy was developed to reduce the proliferation and migration ability of gastric cancer cells. This study provided a more experimental basis for further understanding the mechanism of gastric cancer occurrence and development.

## MATERIALS AND METHODS

### Bioinformatics assay

Perl and R software were used to perform bioinformatics analysis of patient expression and clinical data in the TCGA database (https://portal.gdc.cancer.gov). The protein-protein interaction (PPI) network was constructed by STRING (https://string-db.org/) [19]. The Cytoscape software and its apps were used to display the results [20].

### Cell lines

Human gastric cancer cell lines AGS, MGC-803, SGC-7931, human gastric mucosal epithelial cell line GES1 and human kidney epithelial cell line 297T were purchased from the Cell Bank of the Chinese Academy of Sciences (China) and preserved by the Wuhan University of Science and Technology. All cell lines were cultured in a 5% CO_2_ incubator at 37°C. The culture medium, PBS, trypsin and penicillin/streptomycin were purchased from GIBCO (GIBCO, USA), and fetal bovine serum was purchased from Hyclone (Hyclone, USA).

### Plasmid

The plasmids were deposited by the Wuhan University of Science and Technology. Gag-pol, VSVG, pLVX and pLKO.1 plasmid were used for lentiviral packaging and pmirGLO plasmid was used for the luciferase experiment. Construction and sequencing of plasmids were entrusted to Sangon Biotech (Sangon Biotech, China).

### CCK-8 assay

Logarithmically growing target cells were collected and seeded into 96-well plates (1000 cells per well). After the cells adhered, prepare the CCK-8 working solution described in the instructions and add it to the 96-well plate. After incubation in the incubator, the absorbance of cells at 450 nm was measured using a multi-plate reader (Thermo Fisher Scientific, USA). Each group set up five sub-wells, and three independent experiments were performed. The kit used was CCK-8 Cell Counting Kit (Vazyme, China).

### EdU assay

The logarithmically growing target cells were collected and seeded into 35 mm glass bottom dishes (Biosharp, China) (500 cells were seeded per well). Subsequently, cells were incubated with Edu by adding EdU to the culture medium for 2 h. After incubation the confocal microscope (Olympus, Japan) was used to photograph them, and Image J was used to count EdU-positive cells. In this experiment, more than 50 cells in the field of view were randomly selected for counting, and statistical analysis was performed from five different regions, and three independent experiments were carried out. BeyoClick™ EdU Cell Proliferation Detection Kit (Beyotime Biotechnology, China) was used for the EdU assay.

### Scratch wound healing assay

Logarithmically growing target cells were collected and seeded into 6-well plates (10 000 cells per well). After the cells adhered, a 200 μL pipette tip was used to perform a linear scratch in a 6-well plate. An inverted microscope (Olympus, Japan) was used to record images at the beginning and end of the experiment, and Image J was used to calculate the area of the scratch wound. Three independent experiments were performed in each experimental group.

### Colony formation assay

Logarithmically growing cells of interest were collected and seeded into 6-well plates (500 cells per well) and cultured in an incubator for 14 days. After the incubator culture was completed, the original medium was discarded. The 4% paraformaldehyde (Meilunbio, China), 0.1% crystal violet staining solution (Meilunbio, China) and PBS were used to fix, stain, and wash. Subsequently, the 6-well plate was placed in a fume hood to dry. Colonies larger than 50 cells were counted. Three independent experiments were performed in each experimental group.

### Cell adhesion ability assay

Matrigel (BD Biosciences, USA) was plated in 6-well plates and incubated overnight at 4°C. The logarithmically growing target cells were then collected and seeded into a 6-well plate covered with Matrigel (5 000 cells per well) and incubated in an incubator for 30 minutes. Subsequently, the medium was collected, and an automatic cell counter (Countstar, China) was used to count the non-adherent cells. The number of adhered cells = total number of cells − number of non-adherent cells. Three independent experiments were performed in each experimental group.

### Transwell assay

Transwell chambers (Corning, USA) were used to measure the invasive ability of cells. The logarithmically grown serum-free cultured target cells were collected and seeded into the upper chamber of Transwell (2000 cells per well). In the lower chamber of the Transwell, a medium containing 10% FBS was added. After 48 hours in the incubator (37°C, 5% CO_2_), the original medium was discarded, and 4% paraformaldehyde, 0.1% crystal violet staining solution and PBS were used to fix, stain, and wash. It was then placed in a fume hood to dry, and an inverted microscope was used to record images at the end of the experiment. Two additional replicates were set up for each group of experiments, and three independent experiments were performed in each experimental group.

### Western blot (WB) assay

As described in the instructions, the RIPA method was used to extract proteins from cells or tissues of interest. The BCA method was used to determine the content of the extracted protein. The reagents and kits used were RIPA lysis buffer (Beyotime Biotechnology, China), the protease inhibitor (Beyotime Biotechnology, China), the phosphatase inhibitor (Beyotime Biotechnology, China), and the BCA protein concentration assay kit (Beyotime Biotechnology, China). Proteins were separated according to molecular weight by SDS-PAGE, transferred to PVDF membranes (Membrane, Switzerland), and blocked for 2 hours at room temperature. Subsequently, the PVDF membrane was incubated with the diluted primary antibody at 4°C overnight. After the incubation, the PVDF membrane was incubated with the diluted secondary antibody for 2 hours at room temperature. Finally, ECL combined with the ChemiDoc XRS+ system (BIO-RAD, USA) was used to detect protein expression. Three independent experiments were performed in each experimental group. The reagents and antibodies used were as follows, PAGE gel rapid preparation kit (Yeasen, China), pre-stained protein marker (Yeasen, China), rapid blocking solution (Yeasen, China), primary antibody dilution (Beyotime Biotechnology, China), secondary antibody diluent (Beyotime Biotechnology, China). Anti-GRB10 antibody (1:500) (CST, USA), Anti-β-Actin antibody (1:5000) (Abclonal, China).

### qRT-PCR assay

As described in the instructions, RNeasy Micro Kit and RNeasy Mini Kit (Qiagen, Germany) were used to extract RNA from cells or tissues. HiScript III 1st Strand cDNA Synthesis Kit (Vazyme, China) was used to reverse transcribe RNA to cDNA. miRNA Universal SYBR qPCR Master Mix and 2× ChamQ SYBR Color qPCR Master Mix (Vazyme, China) were used to perform qRT-PCR. Two additional sub-wells were set up per group for qRT-PCR and three independent experiments were performed in each experimental group. The primers were as follows, GRB10 F: 5′-ACCACGGGCTCTGCATAAAG-3′, GRB10 R: 5′-ACGTCCTGGTTTGCTCGTC-3′, β-Actin F: 5′-CTCCCTCACAACAACCGC-3′, β-Actin R: 5′-TACCAGGAACTTCCATACCAAC-3′. Stem-loop method was used to detect the expression of miR-379-5p. The sequence was designed and synthesized by RiboBio (RiboBio, China).

### Dual-luciferase reporter gene assay

The pmiRGLO plasmid was used to construct a dual-luciferase reporter plasmid for the 3′UTR of GRB10. Logarithmically growing target cells were collected and seeded into 96-well plates (1000 cells per well). After the cells adhered, the dual-luciferase reporter plasmid was transfected into the cell line. After 48 hours, the Dual-Luciferase Reporter Assay System (Promega, USA) was used to analyze the relative luciferase activity in the cell line as described in the instructions. Two additional sub-wells were set up in each group, and three independent experiments were performed in each experimental group.

### Tumor formation in nude mice

BALB/C nude mice were purchased from Weitong Lihua Limited Company (China). All nude mice used in this study were housed in the Experimental Animal Center of Wuhan University of Science and Technology. This study was approved by the Ethics Committee of the Wuhan Institute of Life Science and Health. The logarithmic growth target cells suspension was collected and inoculated subcutaneously on the back of nude mice. The weight and tumor size of the nude mice were recorded every 7 days. Nude mice were euthanized 35 days after inoculation with cells, and the subcutaneous tumor tissue was isolated.

### Clinical samples

The study was approved by the Ethics Committee of Tianyou Hospital Affiliated to Wuhan University of Science and Technology. Patients who were treated in the hospital’s surgery from 2017 to 2022 were selected as the research subjects, and the included patients met the diagnostic criteria for gastric cancer. Coagulation and hematopoietic functions were normal. No other tumors merged. All patients were treated by surgical resection, and none of them received radiotherapy and chemotherapy before surgery. All patients gave informed consent to this study, and the experiment was performed according to the Declaration of Helsinki.

### Immunohistochemistry assay

The collected tissue samples were fixed, transparent, embedded and sectioned in sequence. The sections were then dewaxed and hydrated, antigen retrieved, endogenous peroxidase inactivated, blocked, incubated with primary antibody, incubated with secondary antibody, developed with DAB, stained with hematoxylin, reversed with hematoxylin, dehydrated, and mounted. An inverted microscope was used to record immunohistochemical images. Stained sections were examined by a double-blind-based procedure. The IHC score was calculated by multiplying the ratio score (percentage of positively stained cells) by the staining intensity score. The scale score ranges from 0 to 4. Each pathological tissue sample section is given a final score by evaluating 5 high-power fields of densely cellular areas. The reagents and antibodies used were as follows, tissue fixative (Servicebio, China), immunohistochemistry kit (Servicebio, China), and neutral resin (Servicebio, China), Anti-GRB10 antibody (1:500) (Abclonal, China), Anti-Ki67 antibody (1:500) (Abclonal, China).

### Transfection

Logarithmically growing target cells were collected and seeded into 6-well plates (10 000 cells per well). After cells were attached, PEI and Lipofectamine^®^ 3000 (Thermo Fisher Scientific, USA) were used as transfection reagents to transfect plasmids or RNAs into cell lines. Forty-eight hours after transfection, cells were harvested for further experimental analysis.

### Lentiviral infection

Three plasmid lentiviral packaging system was used to construct stably transfected cell lines. Briefly, the HEK293T cell line was transfected according to the mass ratio of the lentiviral plasmid: GAG-POL plasmid: VSVG plasmid 4:3:1. 72 hours after transfection, viral stocks were collected and concentrated using PEG8000. Subsequently, the virus solution was added to the target cells to infect for 24 hours. After infection, stable cell lines were selected using puromycin.

### RNA immunoprecipitation (RIP) assay

RNA immunoprecipitation kit (Geneseed, China) was used to perform RIP experiments as instructed. Briefly, logarithmically growing cells were collected. The collected cells were lysed in the lysis buffer provided by the kit. Lysates were then incubated overnight with magnetic beads coupled to Ago2 (Abclonal, China). IgG served as a control. After incubation, washing and purification were performed in sequence, and the expression of target RNA was detected by qRT-PCR.

### Statistical analyses

Statistical analyses were performed using unpaired two-tailed Student *t*-test and chi-squared test, as well as ANOVA followed by Dunnett. Three levels of significance were used, where ^*^p < 0.05, ^**^p < 0.01, ^***^p < 0.001.

### Availability of data and materials

All data generated or analyzed during this study are included in this published article.

## RESULTS

### Expression and clinical value of GRB10 in cancer

**Figure 1:**
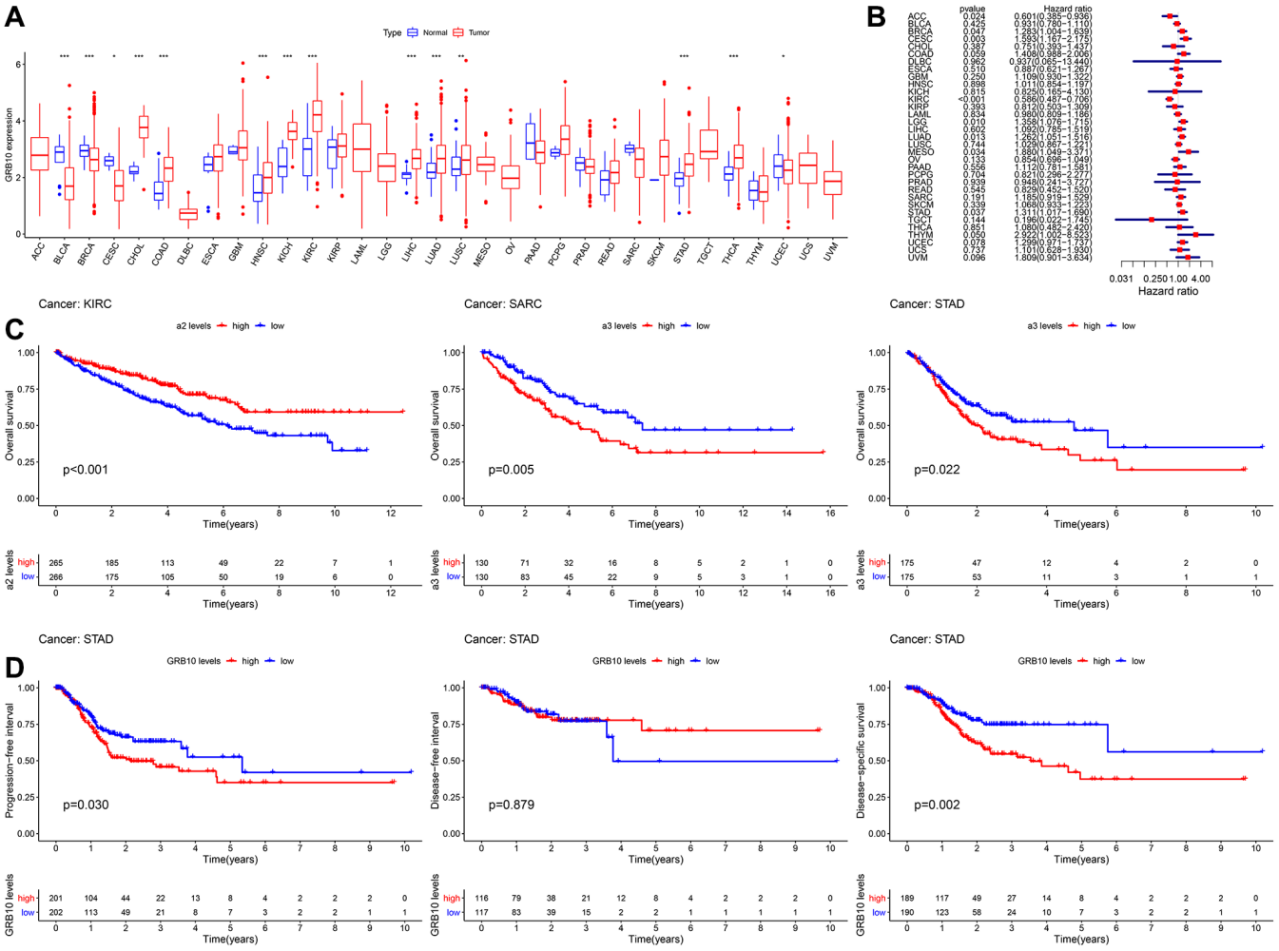
**Expression and clinical value of GRB10 in cancer.** (**A**) Differences in the expression of GRB10 in 33 types of cancer in the TCGA database. Blue represents normal tissue and red represents tumor tissue. (**B**) Univariate Cox analysis of OS in 33 types of cancer patients by forest plots. (**C**) Kaplan-Meier survival curves for OS of KIRC, SARC and STAD. (**D**) Kaplan-Meier survival curves for PFI, DFS and DSS in STAD patients. ^*^p < 0.05, ^**^p < 0.01, ^***^p < 0.001.

We aimed to analyze the expression of GRB10 in cancer. Since there was tissue specificity in the expression of GRB10. Therefore, we collected the mRNA expression profile data of 33 cancers in the TCGA database for analysis, and the result is shown in Figure 1A. In ACC, DLBC, LAML, LGG, MESO, OV, TGCT, UCS and UVM, there was no normal tissue GRB10 expression data. In the other 24 cancers, the expression of GRB10 was up-regulated in most cancers, including CHOL, COAD, HNSC, KICH, KIRC, LIHC, LUAD, LUSC, STAD, and THCA. Notably, GRB10 expression was decreased in BLCA, CECS, UCEC and BRCA. This result suggests that the function of GBR10 may be tissue-specific.

Next, evaluated the clinical value of GRB10 in 33 cancer species. The results of Cox univariate analysis of the relationship between the expression of GRB10 and overall survival (OS) are shown in Figure 1B, 1C. GRB10 play different functions in different cancer types. In KIRC and ACC, low expression of GRB10 was significantly associated with poorer OS. However, in CESC, LGG, LUAD, MESO, STAD, BRCA and THYM, high expression of GRB10 was significantly associated with poorer OS. Furthermore, KM analysis revealed that the expression of GRB10 was significantly associated with OS only in KIRC, SARC and STAD. The above results suggested that the expression of GRB10 was an important factor affecting the survival of STAD and KIRC cancers, although their relationship would vary by tumor type.

Analysis of data from the TCGA database showed altered expression of GRB10 in most types of cancer (Figure 1A). Notably, the results were shown in Figure 1B–1D. Only in gastric cancer, GRB10 expression was found to correlate with OS, progression free survival (PFI), disease free survival (DFI) and disease specific survival (DSS). The above results showed that GRB10 was involved in the occurrence and development of gastric cancer. As we noted in the Introduction, GRB10 has not been reported for gastric cancer. Therefore, we would focus on the relationship between the expression of GRB10 and gastric cancer.

### The relationship between GRB10 expression and clinicopathological features in gastric cancer

**Figure 2:**
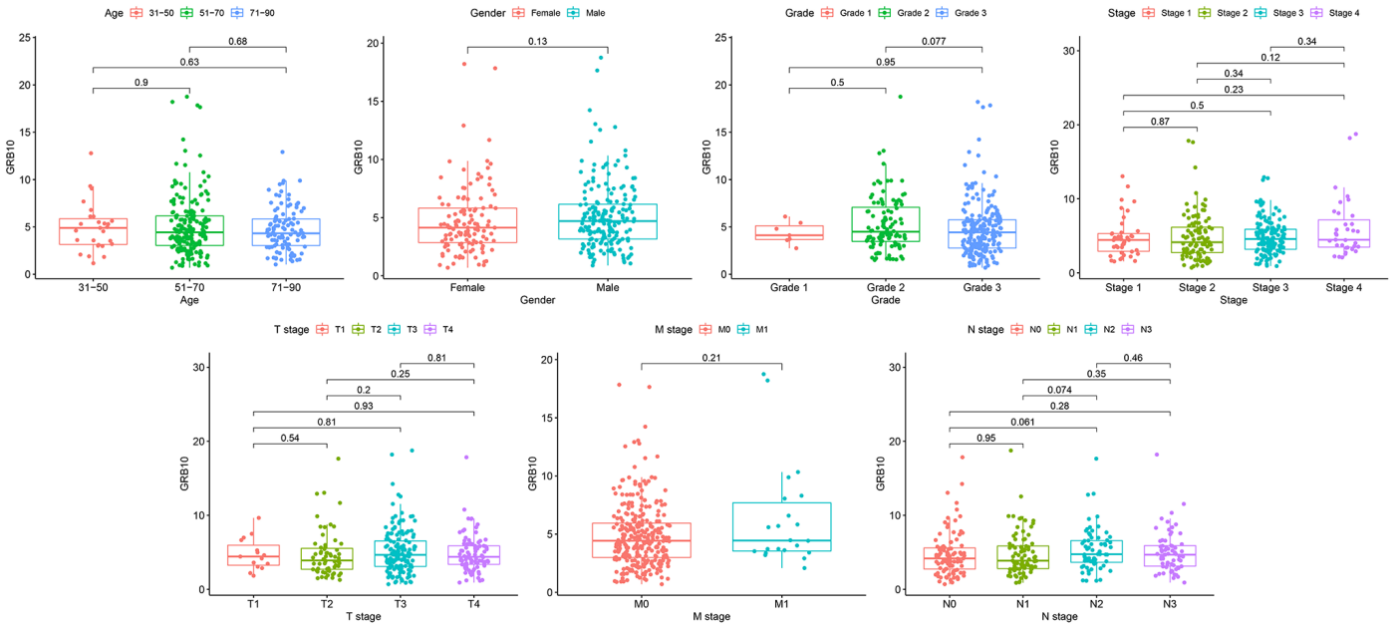
The relationship between GRB10 expression and clinicopathological features in gastric cancer.

Clinical information of gastric cancer patients from the TCGA database was downloaded for analysis. After removing data with incomplete clinical information, the clinical features of GRB10 expression were analyzed. The results are shown in Figure 2. The expression of GRB10 was not significantly correlated with age and gender. At the same time, its expression was also not significantly correlated with pathological grade, grade, stage, T stage, M stage and N stage. Combined with the results of Figure 1A. It showed that the expression of GRB10 was increased in the tumor tissue of gastric cancer patients. This increased expression was unrelated to the patient’s age, gender and pathological grade. Therefore, it was expected to be used as a tumor marker for gastric cancer.

### GO and KEGG functional enrichment

**Figure 3:**
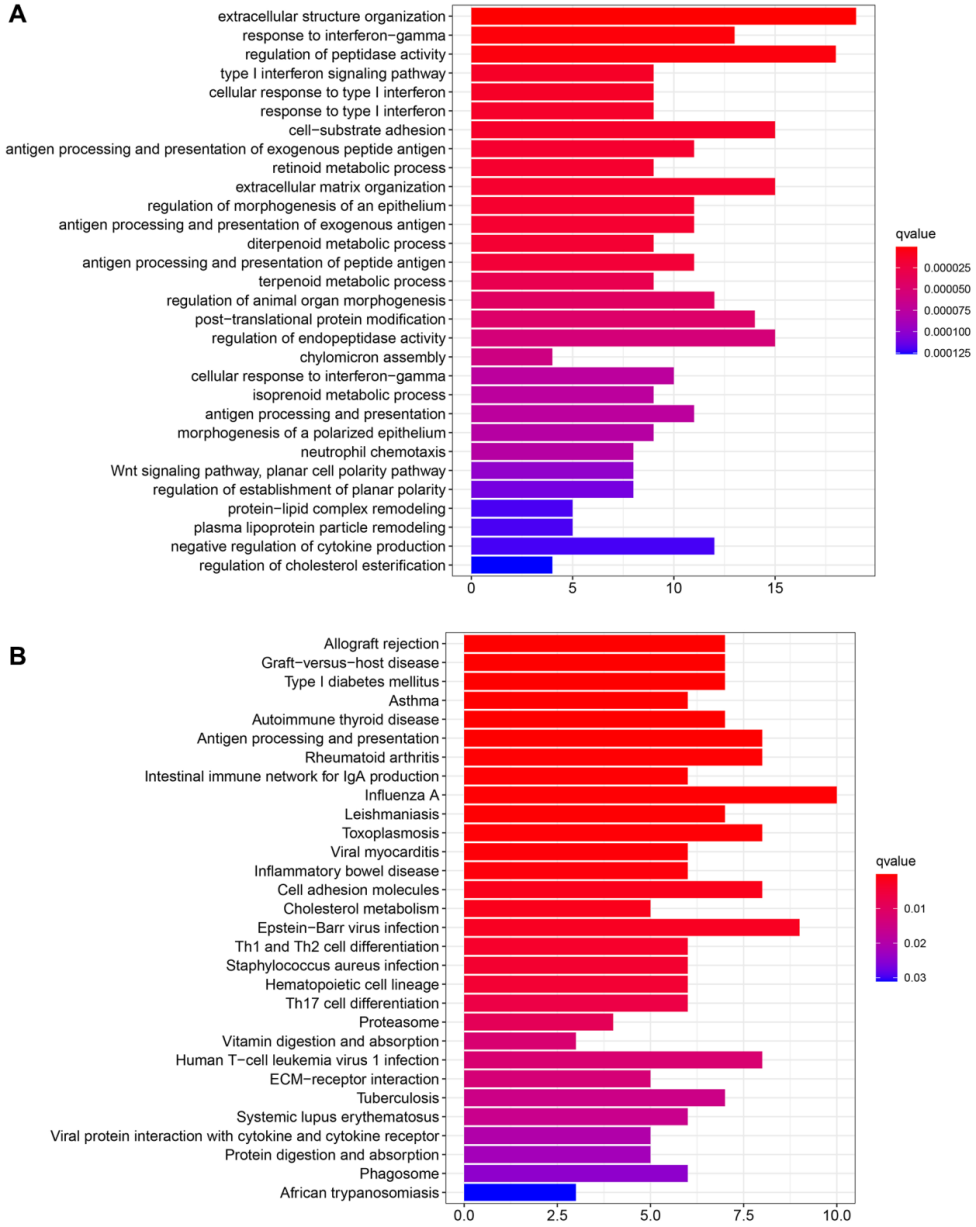
**Functional enrichment of GRB10.** (**A**) Functional enrichment was analyzed via GO. (**B**) Functional enrichment was analyzed via KEGG.

R software was used to perform GO and KEGG functional enrichment. The top 30 functions and pathways were displayed in descending order of the corrected *P* values. The results are shown in Figure 3A, 3B. The top five GO functional enrichments were extracellular structure organization, response to interferon-gamma, regulation of peptidase activity, type I interferon signaling pathway and cellular response to type I interferon. The top five KEGG enrichments were Autoimmune thyroid disease, Asthma, Type I diabetes mellitus, Graft−versus−host disease and Allograft rejection. Among them, the seventh cell-substrate adhesion molecules enriched by GO and the seventeenth cell adhesion molecules enriched by KEGG attracted our attention. It was well known that cell adhesion was the most basic life activity of cells and plays a key role in the cell proliferation and migration process [21]. The above enrichment of bioinformatics functions suggested that GRB10 was involved in regulating gastric cancer progression.

### Protein interaction network

**Figure 4:**
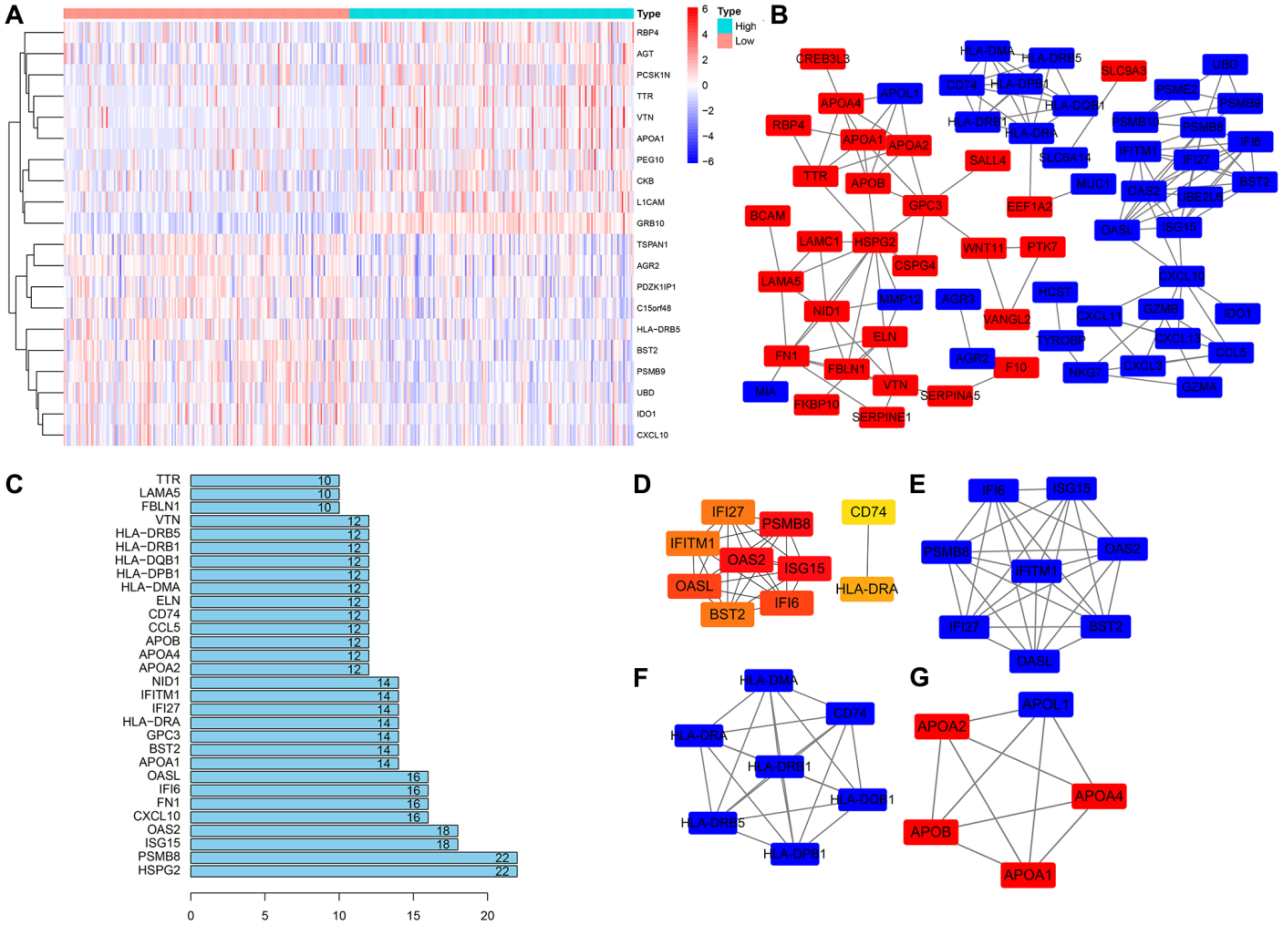
**Construction of the PPI network and module analysis of GRB10.** (**A**) The heatmap of top 10 co-expressed genes with GRB10. (**B**) PPI network was constructed using Cytoscape software. (**C**) The top 30 nodes in the number of edges of the protein-protein interaction network node from the string database. (**D**) The hub genes were identified using Cytoscape. (**E**–**G**) The core sub-network of the protein-protein interaction network.

According to the expression of GRB10, it was divided into high expression group and low expression group, and R software was used to screen the genes co-expressed with GRB10. The top 10 genes are shown in Figure 4A, and a total of 150 genes co-expressed with GRB10 were screened, of which 77 were up-regulated and 73 were down-regulated. These genes were imported into the String tool, their potential interactions were analyzed, and Cytoscape software was used to display the results. After hiding the disconnected nodes, the results are shown in Figure 4B, 4C. The top five nodes of the edges of the node were HSPG2, PSMB8, ISG15, OAS2 and CXCL10. At the same time, the Cytohubba app in Cytoscape software was used to analyze the hub genes of these co-expressed genes. The results are shown in Figure 4D. The top 5 genes were PSMB8, OAS2, ISG15, OASL and IFI6. Furthermore, the MCODE app was used to analyze the functional modules of these co-expressed genes. The top 3 sub- networks are shown in Figure 4E–4G. The above bioinformatics analysis was helpful in determining the specific biological function of GRB10 in gastric cancer.

### Higher GRB10 expression in gastric cancer cell lines and tissues

**Figure 5:**
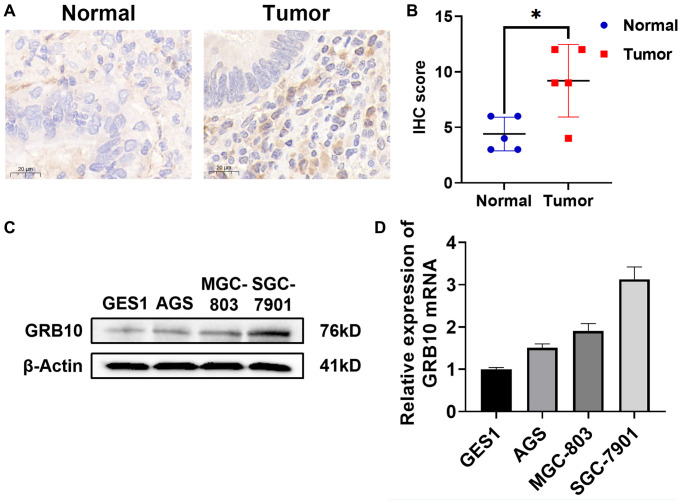
**Expression of GRB10 in human gastric cancer cell lines and tissues.** (**A**) Typical immunohistochemistry of GRB10 in gastric cancer samples. (**B**) IHC staining score of GRB10 in gastric cancer samples. (**C**) The expression of GRB10 in gastric cancer cell lines was detected by Western Blot. (**D**) The relative expression of GRB10 mRNA in gastric cancer cell lines was detected by qRT-PCR.

To investigate whether the expression of GRB10 was consistent with the results predicted in the database. We used immunohistochemistry to evaluate the expression of GRB10 in collected 6 gastric cancer patients and paracancerous tissue specimens. The results showed that the expression of GRB10 in gastric cancer tissues was significantly higher than that in adjacent tissues (Figure 5A, 5B). In addition, we also confirmed the expression of GRB10 by cell lines. As shown in Figure 5C, 5D, GRB10 expression was elevated in human gastric cancer cell lines AGS, SGC-7901 and BGC-803 compared with human gastric mucosal cell line GES1. It was worth noting that in the human gastric cancer cell line AGS cell line, although the results of qRT-PCR showed that the expression of GRB10 was higher than that of GES1, the results of Western Blot did not.

### GRB10 promotes the proliferation and migration ability of gastric cancer cells *in vitro*

**Figure 6:**
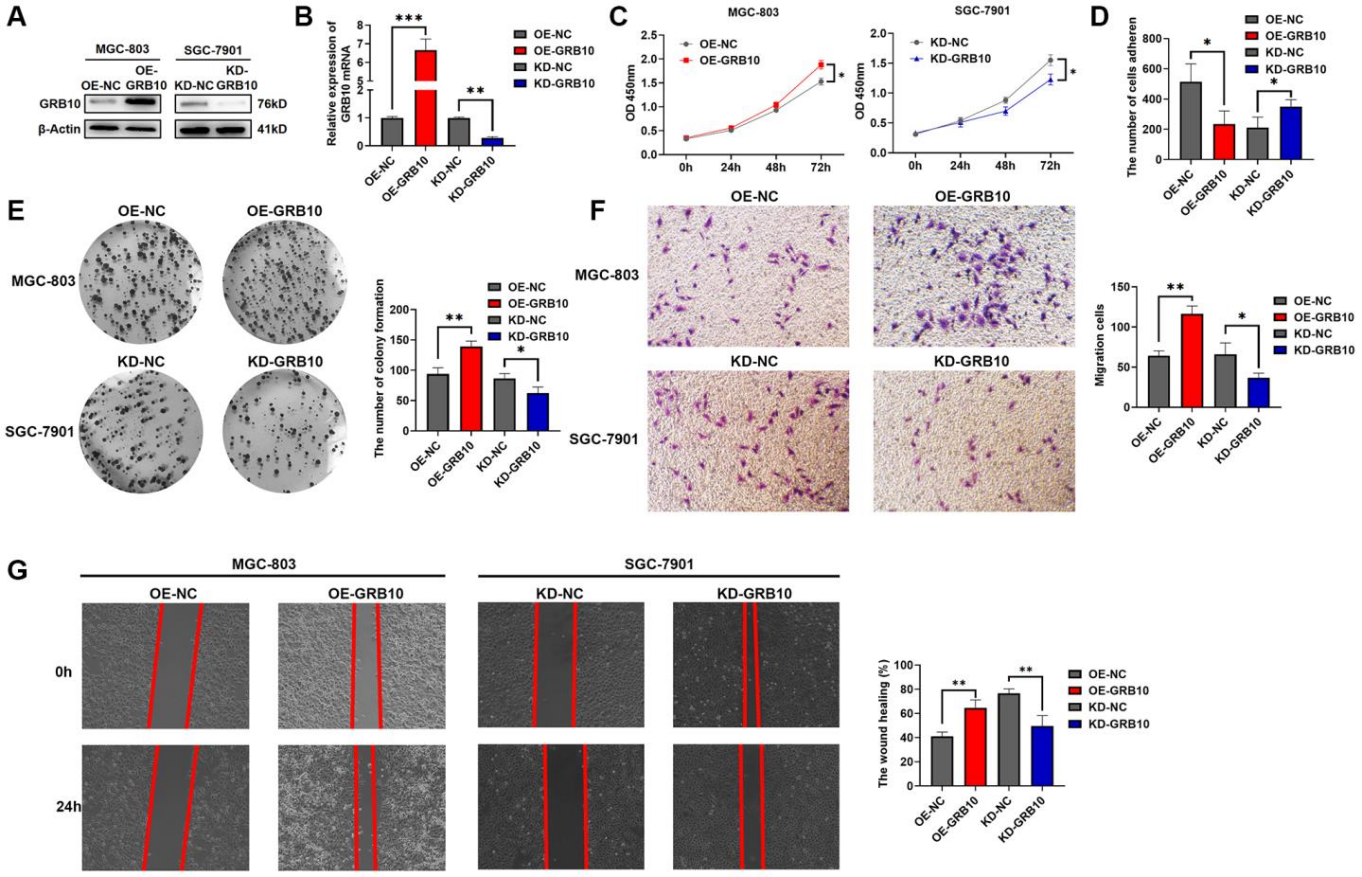
**GRB10 promoted gastric cancer cell proliferation and migration ability.** (**A**) Verification of GRB10 knockdown and overexpression was detected by Western Blot. Abbreviations: OE: overexpression group; KD: knockdown group. (**B**) Verification of GRB10 knockdown and overexpression was detected by qRT-PCR. (**C**) Cell proliferation was determined by the CCK-8 assay. (**D**) Effects of GRB10 on cell adhesion ability. (**E**) Cell proliferation was determined by the colon formation assay. (**F**) Cell migratory ability detected by Transwell migration assay. (**G**) Cell migratory ability detected by scratch wound healing assay. ^*^p < 0.05, ^**^p < 0.01, ^***^p < 0.001.

Among the three gastric cell lines analyzed, we stably constructed GRB10-knockout SGC-7901 and GRB10-overexpress MGC-803 cell lines stably using lentiviral packaging technology. The results of knockdown and overexpression efficiencies are shown in Figure 6A, 6B. Meanwhile, based on the above bioinformatics analysis, the expression of GRB10 may be involved in regulating the progression of gastric cancer. The effect of changes in expression of GRB10 on the proliferation ability of gastric cancer was analyzed by CCK-8, clone formation assay. The results are shown in Figure 6C, 6E. Knockdown of GRB10 significantly reduced the proliferation ability of gastric cancer cells, while overexpression of GRB10 increased the proliferation ability of gastric cancer cells. Similarly, the results of scratch healing assay, Transwell assay and adhesion assay also showed that cell migration ability increased with the expression of GRB10. However, cell adhesion ability decreased with the expression of GRB10 (Figure 6D, 6F, 6G).

### Knockdown of GRB10 reduces gastric cancer progression *in vivo*

**Figure 7:**
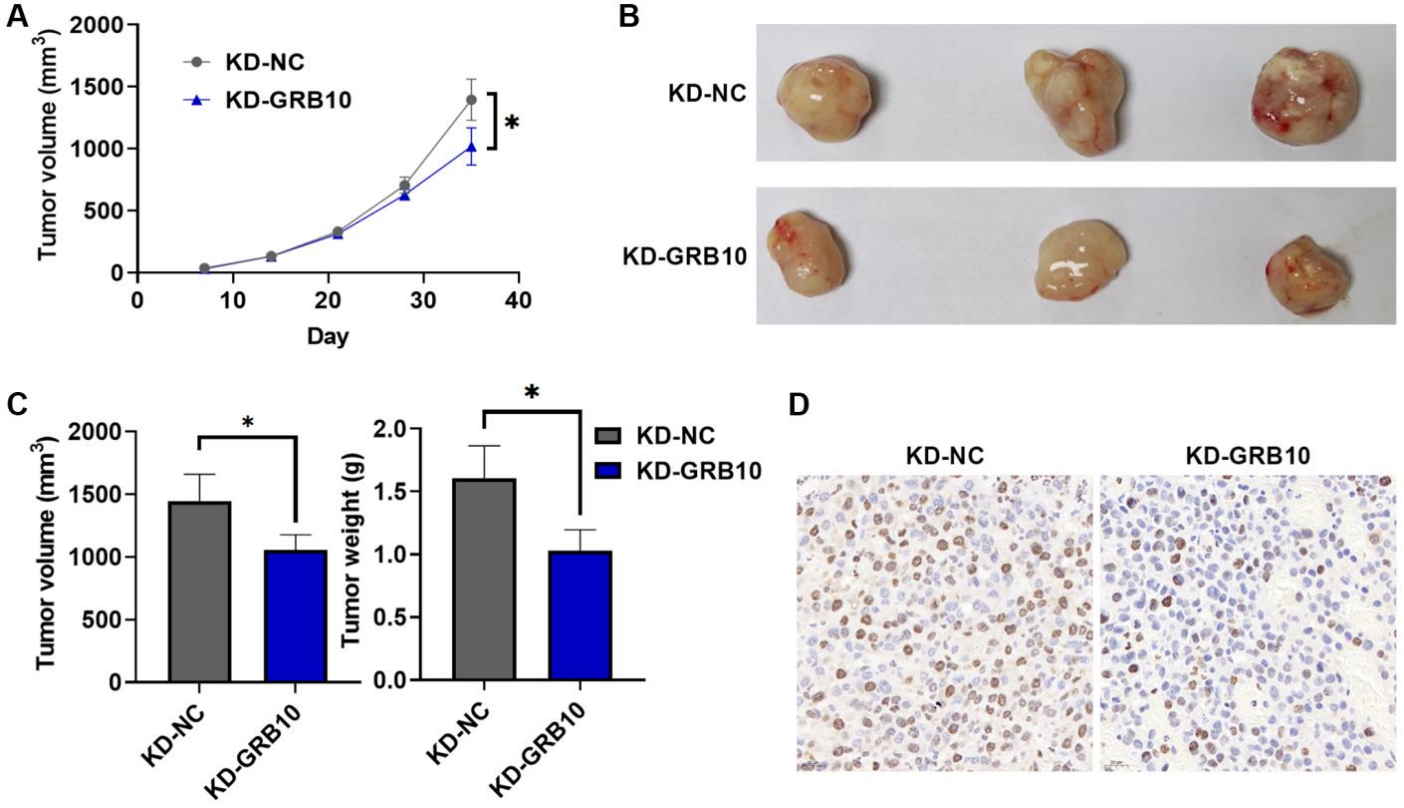
**GRB10 knockdown inhibited tumor growth *in vivo*.** (**A**) Tumor growth curve. Abbreviation: KD: knockdown group. (**B**) Representative pictures of subcutaneous tumors in each group. (**C**) Tumor volume and weight in a subcutaneous tumor model. (**D**) Ki67 immunohistochemical staining. ^*^p < 0.05, ^**^p < 0.01, ^***^p < 0.001.

To further explore whether the expression of GRB10 was also involved in the regulation of gastric cancer progression *in vivo*, the SGC-7901 cell line stably knocked down GRB10 and its control were injected subcutaneously into nude mice. At the same time, the volume of subcutaneous tumor tissue in nude mice was recorded every 3 days, and nude mice were sacrificed 35 days after inoculation with cells. The results are shown in Figure 7A. Knockdown of GRB10 significantly reduced the growth rate of subcutaneous tumors compared with the control group. These subcutaneous tumor tissues were isolated for further study. Knockdown of GRB10 significantly reduced subcutaneous tumor weight and volume (Figure 7B, 7C). Meanwhile, IHC results also showed that the expressions of Ki67 in the low GRB10 expression group were also lower than those in the high GRB10 expression group (Figure 7D). The above results showed that the knockdown of GRB10 inhibited tumor growth *in vivo*.

### miR-379-5p reduces the proliferation and migration ability of gastric cancer by targeting GRB10

**Figure 8:**
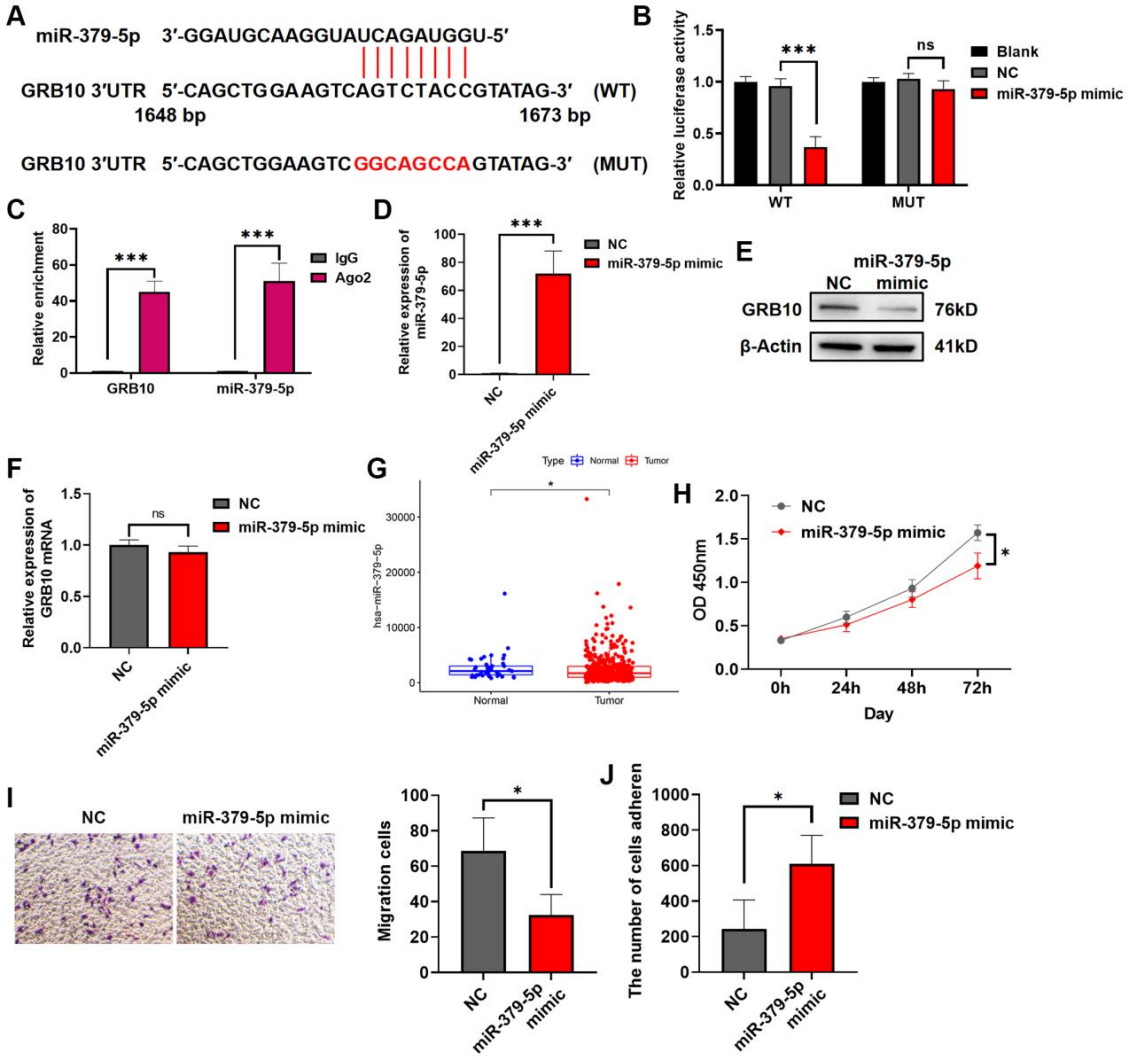
**miR-379-5p reduces the proliferation and migration ability of gastric cancer by targeting GRB10.** (**A**) Diagram of the GRB10 mRNA and miR-379-5p binding site. Abbreviation: KD: knockdown group. (**B**) The relative luciferase activity was measured with a dual-luciferase reporter assay. (**C**) RIP assay was conducted using an antibody against Ago2. (**D**) The relative expression of miR-379-5p was detected by qRT-PCR. (**E**) The expression of GRB10 was detected by Western Blot. (**F**) The relative expression of GRB10 mRNA was detected by qRT-PCR. (**G**) MiR-379-5p expression in the TCGA database. (**H**) Cell proliferation was determined by the CCK-8 assay. (**D**) Effects of miR-379-5p on cell adhesion ability. (**I**) Cell migratory ability detected by Transwell migration assay. (**J**) Effects of miR-379-5p on cell adhesion ability. ^*^p < 0.05, ^**^p < 0.01, ^***^p < 0.001.

As mentioned in the introduction, miRNA was an endogenous non-coding RNA of about 22 nt, which regulated gene expression by targeting mRNA. To search for miRNAs upstream of GRB10, we searched for potential GRB10-regulated miRNAs by bioinformatics. The results are shown in Figure 8A. There was a potential binding site for miR-379-5p on the 3′UTR of GRB10. Therefore, it was verified by luciferase assay and RIP experiment. The results are shown in Figure 8B, 8C. After transfection of miR-379-5p mimic in gastric cancer cell lines. It significantly reduced the activity of GRB10 WT luciferase, but did not alter the luciferase activity of GRB10 MUT whose binding sites were mutated. Furthermore, overexpression of miR-379-5p in gastric cell line SGC-7901 significantly reduced the expression of GRB10 protein in cells, but did not reduce its mRNA (Figure 8D–8F). To understand whether miR-379-5p was related to the occurrence and progression of gastric cancer, the expression of miR-379-5p in gastric cancer was analyzed in combination with the TCGA database. Compared with normal tissues, the expression of miR-379-5p was decreased in gastric cancer (Figure 8G). Further experiments showed that overexpression of miR-379-5p in cells significantly reduced gastric cancer cell proliferation and migration ability and restored cell adhesion ability (Figure 8H–8J). On this basis, the rescue experiment was carried out at the same time. After overexpression of GRB10, cell proliferation and migration ability were significantly increased. And overexpression of miR-379-5p mimic in cells significantly reduced the increase of cancer malignancy caused by GRB10 upregulation (Supplementary Figure 1). Taken together, these results demonstrated that miR-379-5p reduced GRB10 expression by targeting its 3′UTR. Moreover, overexpression of GRB10 reduced the proliferation and migration ability of gastric cancer cells and restored the adhesion ability.

## DISCUSSION

Gastric cancer is one of the common malignant tumors which seriously threatens human health [1]. Although the level of medical technology continues to improve, the diagnosis and treatment of gastric cancer are still not optimistic, and the 5-year overall survival rate is less than 30% [2]. Cancer cell metastasis is the main reason affecting the curative effect of gastric cancer [22, 23]. Therefore, actively exploring the mechanism of gastric cancer metastasis, selecting appropriate diagnostic markers, and discovering new therapeutic targets are crucial for improving the survival rate of gastric cancer patients.

In previous studies, GRB10 was thought to be mainly involved in the regulation of mammalian growth and development [24, 25], gastrulation [26, 27], and blood sugar regulation [28]. However, recent studies have shown that GRB10 was abnormally expressed in various cancers, and changes in its expression were closely related to tumor formation and progression [29–31]. In leukemia patients, Kazi et al. [14] found that the expression of GRB10 was elevated and that elevated expression of GRB10 was associated with FLT3-ITD-mediated cell survival. In addition, in glioma, the study by Chen et al. [32] found that GBR10 is expected to serve as a novel glioma tumor-promoting gene. Knockdown of GRB10 inhibited cells from the S phase to the G2/M phase and reduced cell proliferation. Further biological information analysis found that the expression of GRB10 was positively correlated with the EMT system pathway. Similarly, Khan et al. [33] showed that GRB10 plays a tumor-promoting role in prostate cancer. However, the relationship between the expression of GRB10 and the occurrence and development of gastric cancer has not been reported yet. In this study, the expression of GRB10 in 24 types of cancers in the TCGA database was analyzed. The expression of GRB10 was found to be elevated in 10 types of cancer. Combined with clinical information analysis found. Not only was the expression of GRB10 elevated in gastric cancer, but high expression of GRB10 was significantly associated with poorer overall prognosis survival. Further studies revealed that the knockdown of GRB10 reduced the proliferation of gastric cancer cells. In addition, the study of cell migration also showed the same result. The above data indicated that GRB10 was involved in regulating the occurrence and development of gastric cancer.

miRNA was a kind of non-coding RNA widely distributed in the human body. It regulated gene expression by combining with the complementary site of 3′UTR to destroy its transcriptional stability [34, 35]. Its expression regulated the expression of a variety of genes, which was crucial to the occurrence and development of gastric cancer [36, 37]. In previous studies, miR-379-5p was overexpressed in most tumors. And overexpression of miR-379-5p in breast cancer cell lines and endometrial cancer cell lines significantly reduced the proliferative ability of cancer cell lines [38, 39]. In this study, the expression of miR-379-5p was also found to be lower in gastric cancer. Restoring the expression of miR-379-5p in cells reduced the proliferation and migration ability of gastric cancer cells. At the same time, it was found that miR-379-5p negatively regulated the expression of GRB10 protein, but did not affect its mRNA expression. Sequence analysis revealed a potential binding site for miR-379-5p on the 3′UTR of GRB10. Further experiments with a luciferase reporter gene also confirmed that miR-379-5p regulates the expression of GRB10 by targeting this site.

In conclusion, in this study, it was revealed that the expression of GRB10 is closely related to the proliferation and migration ability of gastric cancer cells. These findings provided a potential rationale for developing a therapeutic approach based on its expression. However, this study also had some limitations. For example, only the expression of GRB10 was analyzed in the TCGA database, in which the patient samples were mostly white, and there were fewer samples from black and yellow patients. In the follow-up work, more samples will be collected to analyze further the relationship between GRB10 expression and gastric cancer patients of different ethnicities. In addition, the specific molecular mechanism of GRB10 regulating the proliferation and migration of gastric cancer is not fully understood, and further research is needed.

## Supplementary Materials

Supplementary Figure 1
