# Supplementary material for: GRB10 is a novel factor associated with gastric cancer proliferation and prognosis
Source: Aging (Albany NY). 2023 Mar 23;15(9):3394–409. doi: 10.18632/aging.204603 (PMC10449302; doi:10.18632/aging.204603)
Supplement: Supplementary Figure 1 [file aging-15-204603-s001.pdf]

SUPPLEMENTARY FIGURE

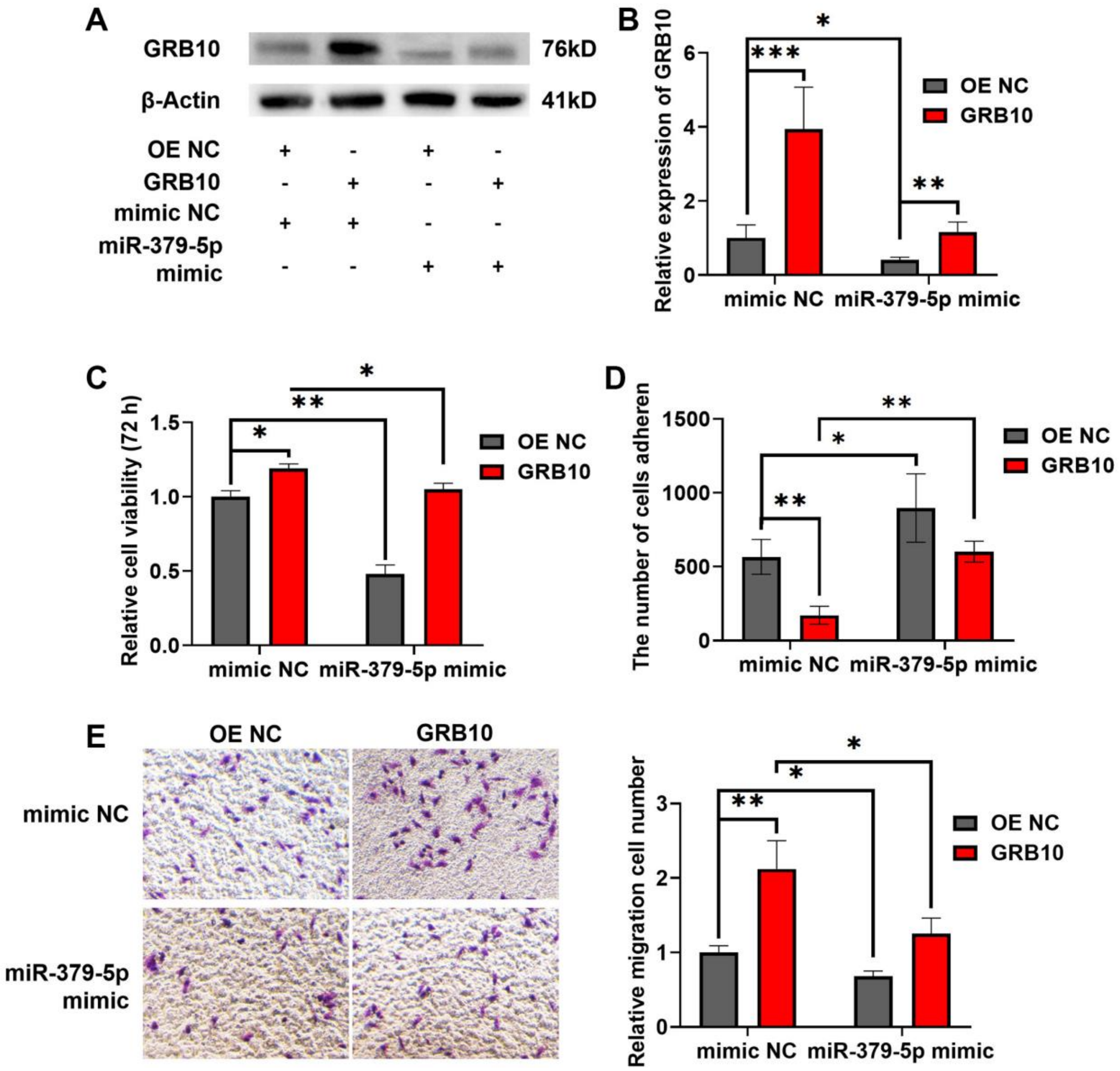

Supplementary Figure 1. MiR-379-5p rescues increased proliferation migration of gastric cancer cells caused by GRB10. (**A**) The expression of GRB10 in gastric cancer cell lines was detected by Western Blot. (**B**) The relative grayscale from WB experiments. (**C**) Cell proliferation was determined by the CCK-8 assay. (**D**) Effects of GRB10 on cell adhesion ability. (**E**) Cell migratory ability was detected by Transwell migration assay.
